# A DIC-Based Study on Compressive Responses of Concrete after Exposure to Elevated Temperatures

**DOI:** 10.3390/ma12132044

**Published:** 2019-06-26

**Authors:** Sheng Xiang, Lei Zeng, Jicheng Zhang, Juan Chen, Yanhua Liu, Guoyuan Cheng, Jinxu Mo

**Affiliations:** School of Urban Construction, Yangtze University, Jingzhou 434023, China

**Keywords:** digital image correlation, full-field, elevated temperature, concrete

## Abstract

This paper provides an experimental investigation on the cracking process and residual mechanical properties of concrete after exposure to elevated temperatures. A total of 36 standard concrete prism specimens were tested after exposure to high temperatures of up to 600 °C. The failure modes, cracking process, residual mechanical properties, deformation characteristics and the strain distribution on the surface during the loading procedure were presented. The influences of exposure temperature and water–cement ratio (w/c) were interpreted. The digital image correlation (DIC) method was applied to quantitatively and visually characterize the development of cracking and relative displacement on the concrete surface. The findings suggest that the residual compressive strength and elastic modulus of the concrete decreases gradually with the increasing temperature, especially in the specimens with lower w/c ratio. The DIC technique provides an effective means to measure very precise and detailed information, including the crack opening and distribution of strain on the concrete surface.

## 1. Introduction

Concrete is one of the most widely used building materials, but it is vulnerable to explosive spalling at elevated temperatures caused by fire, explosion or furnaces nearby [1,2,3,4,5,6,7]. The residual mechanical properties significantly reduce after exposure [8,9,10,11,12,13]. Building fire accidents all over the globe have drawn attention to understanding residual performance at elevated temperatures. Meanwhile, mechanical properties are strongly affected by the cracks in concrete, which directly reflect the damage process and health status of concrete. Therefore, an effective method evaluating the crack propagation process and load-carrying capacity is particularly important.

In the past decades, researchers have focused on the mechanical properties of concrete after exposure to elevated temperatures, such as compressive strength, splitting tensile strength and elastic modulus. Results have indicated that the degree of deterioration depends on the peak temperature, exposure time, cooling system and concrete composition [8,9,14,15]. Omer Arioz et al. [1] carried out experimental tests to investigate concrete after exposure to elevated temperatures from 200 °C to 1200 °C. The residual compressive strength and weight losing rate had a sharp jump when the temperature of the concrete was subjected to 800 °C. The specimens completely decomposed and lost their binding properties after exposure at 1200 °C. Germán Ercolani et al. [14] explored the effects of elevated temperatures and different cooling systems on the mechanical properties of concrete. The test results revealed that the water cooling method caused serious decrease of compressive strength, while the increase of water volume used for cooling aggravated this trend. The micro-cracks of concrete at high temperatures were caused by the different thermal strains of cement matrix and aggregates. Other studies [15,16] revealed that concrete made of calcareous aggregates performed better than concrete manufactured by using siliceous aggregates. Chang et al. [17] proposed a single equation for the complete stress–strain relationship of heated and unheated concrete. The equation was applicable to the experimental curve both in ascending and descending processes.

The research discussed above mostly focused on macro properties using traditional instruments, such as strain gauge, extensometer and displacement sensor. However, there are few studies on micro-damage research reflecting the damage process and the full field strain distribution in concrete. Recognizing the drawback of traditional measurement tools, optical measurement methods have gradually gained the attention of researchers. Holographic interference, electronic speckle interference, moiré interference and digital image correlation (DIC) are several commonly used optical measurement methods. Compared with other optical measurement methods, DIC technology has the advantages of simple optical path and insensitive to vibration. DIC technology is widely used in many fields because of its characteristics such as full-field test, simple operation and low requirement for experimental environment [18,19,20,21,22,23]. These tests are mainly done to study the complex deformation and crack development process of materials and components, which are not available in traditional measurement tools. Since concrete is a composite material composed of a mixture of various materials, the development of deformation and cracks after exposure to elevated temperatures are difficult to obtain with a displacement gauge and strain gauge. For the aim of obtaining the strain developments of the continuous cracking process and crack openings, the DIC technique is used.

In this paper, an experimental investigation on the cracking process and residual mechanical properties of concrete after exposure to elevated temperatures was carried out, and a combination of the DIC technique and mechanical test were proven as a viable approach for the health monitoring of concrete. According to the design codes (e.g., the European standard EN 1992-1-2 (2004) [24]), after exposure to 600 °C, concrete will be heavily damaged and its bearing capacity will have been reduced by more than half. In light of that, the maximum temperature of this test was taken as 600 °C. A total of 36 standard concrete prism specimens were tested after exposure to high temperatures of up to 600 °C. The failure modes, cracking process, residual mechanical properties, deformation characteristics and the strain distribution on the surface during the loading procedure were presented. The influences of exposure temperature and water–cement ratio (w/c) were interpreted.

## 2. Experimental Investigations 

### 2.1. Test Specimens

The guiding ideal of this experiment was to evaluate the behavior of concrete with exposure to elevated temperatures. For this purpose, 36 specimens were designed to investigate the influence of the w/c and the peak temperature on the mechanical properties and cracking process of concrete. The specimens’ variables were the w/c ratio (0.5, 0.42 and 0.34) and the exposure temperature (200, 400 and 600 °C). The specimens were named according to their w/c ratio and exposure temperature. For instance, C-1-200 corresponds to the mixture produced with a 0.5 w/c ratio and exposed to 200 °C. The details of specimens are shown in Table 1.

The specimens were prepared by using common ordinary Portland cement with 28 days strength of 42.5 MPa, local river sand with a fineness modulus of 2.4 and coarse aggregates with a size range of 5–10 mm approximately. The mix design for the concrete to be tested was determined using steps outlined in Specification for mix proportion design of ordinary concrete (JGJ55-2011) [25] and was further refined through multiple trials adjusting the mix to suit slump results. The cement and aggregates were mixed firstly. The water was then added, and the concrete was mixed for 3 min. The concrete specimens were cured for 24 h in plastic molds, and then were de-molded and cured in water for 28 days. To ensure moisture content stability, the specimens were air-dried in a laboratory for about 60 days before heating. 

### 2.2. Heating Procedure and Furnace

**Figure 1:**
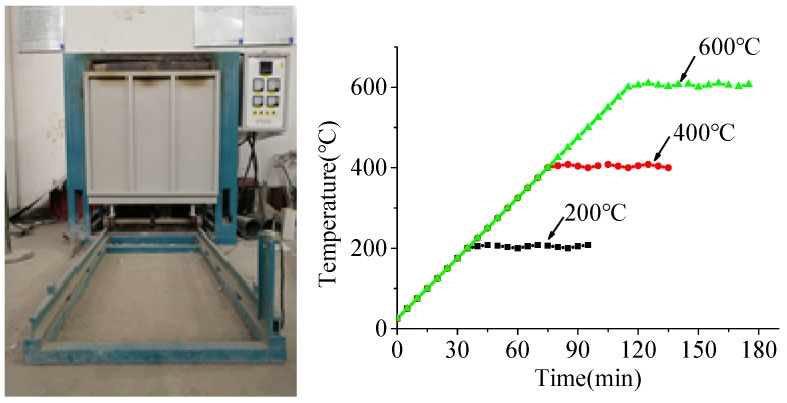
The electrical furnace and temperature recorded by the furnace.

An electrical furnace, 80 cm × 80 cm in cross section and 110 cm in height, was used to simulate the fire situation, as shown in Figure 1. The heating rate was 5 °C/min in this experiment, which was controlled automatically by adjusting the electric current. After heating to the target temperature (200, 400 and 600 °C), the temperature was maintained for 60 min, as shown in Figure 1. Then, the specimens were cooled down naturally and removed from the furnace until the temperature of the furnace chamber decreased to 100 °C to avoid thermal shock caused by a large cooling rate.

### 2.3. Test Setup and Procedure

**Figure 2:**
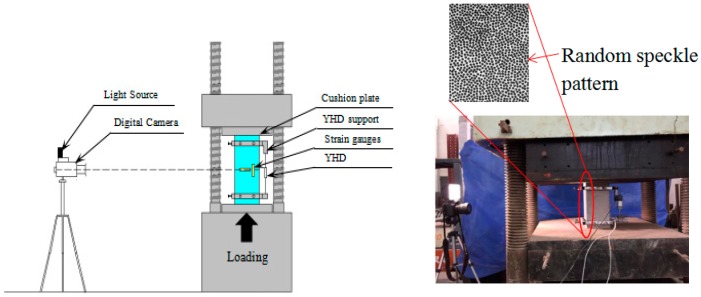
The testing device and instrumentations.

The axial compression tests on heated and unheated specimens were performed using a hydraulic press machine with a maximum ability of 5000 kN and controlled by the actuator displacement at a rate of 0.2 mm/min according to Standard for test method of mechanical properties on ordinary concrete (GB/T 50081-2002) [26]. A YHD displacement meter was attached on the specimen surface to measure the average strain over a 20 cm range in the middle of the specimen. Two strain gauges were attached to the middle of the test piece, one horizontal and the other vertical. In addition, the DIC technique was used to measure the deflections, monitor the development of cracks and detect the strain components. As shown in Figure 2, the camera was mounted on a rigid tripod and the distance between the camera and the surface of the specimen was approximately 1000 mm. An external light source was also provided to ensure a good lighting condition. In order to provide a random gray distribution for the matching process, a random speckle pattern was applied on the surface of the specimens, as shown in Figure 2.

### 2.4. DIC Principles

DIC was originally developed by Peters and Ranson [27] from the USA and Yamaguchi [28] from Japan in the 1980s. Over the past 40 years, DIC has been developed in various aspects by many researchers over the world, and has become an effective and popular technique. During this time, Sutton and colleagues have conducted deep research on the theory and application of DIC technology, which has promoted the wide application of DIC technology [29,30,31,32]. DIC technology is an optical-numerical full-field surface displacement measurement method. It is based on a comparison between two images of the specimens coated by a random speckle pattern in the undeformed and deformed state.

**Figure 3:**
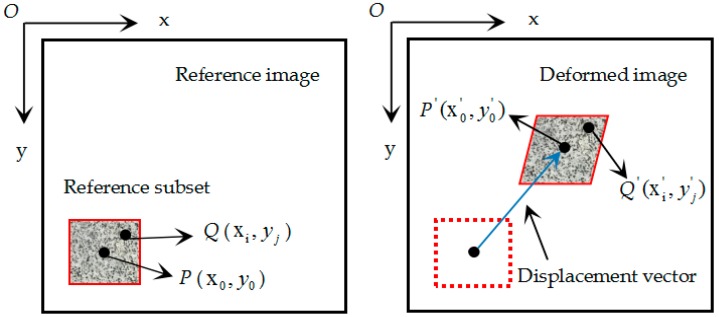
Schematic diagram of in-plane displacement and deformation of sub-regions.

The basic principle of the DIC technique is to search for a maximum correlation between small regions (called subsets or subwindows) of the specimen in the undeformed and deformed states, as illustrated in Figure 3. In order to obtain the displacements of point *P*, a square reference subset centered on point $P(x_{0},y_{0})$ is selected from the reference image to track its corresponding position in the deformed image. A subset of squares is chosen for matching rather than a single pixel, because the subset containing a wider variation in gray levels will distinguish itself from other subsets and can be more uniquely identified from other subsets in the deformed image [33].

In order to assess the similarity degree between the undeformed subset and the deformed subset, a cross-correlation (CC) criterion or sum-squared difference (SSD) correlation criterion was adopted. The matching process was conducted through searching the peak position of distribution of the correlation coefficient. When the extremum of the correlation coefficient was detected, the position of deformed subset was determined. The most popular correlation criteria include the zero-normalized cross-correlation (ZNCC) and zero-normalized sum of squared differences (ZNSSD). These two criterion were highly recommended for practical use, and were theoretically unaffected by changes in illumination, as they were invariant to a linear transformation in gray intensity [34,35]. The correlation criteria $C_{ZNCC}$ and $C_{ZNSSD}$ take the following form:

$$C_{ZNCC}=\frac{\sum_{x=-M}^{M}\sum_{y=-M}^{M}\left[f(x,y)-f_{m}\prod g(x^{\prime },y^{\prime })-g_{m}\right]}{\sqrt{\sum_{x=-M}^{M}\sum_{y=-M}^{M}{\left[f(x,y)-f_{m}\right]}^{2}}\sqrt{\sum_{x=-M}^{M}\sum_{y=-M}^{M}{\left[g(x^{\prime },y^{\prime })-g_{m}\right]}^{2}}},$$

$$C_{ZNSSD}={\sum_{x=-M}^{M}\sum_{y=-M}^{M}\left[\frac{f(x,y)-f_{m}}{\sqrt{\sum_{x=-M}^{M}\sum_{y=-M}^{M}{\left[f(x,y)-f_{m}\right]}^{2}}}-\frac{g(x^{\prime },y^{\prime })-g_{m}}{\sqrt{\sum_{x=-M}^{M}\sum_{y=-M}^{M}{\left[f(x,y)-f_{m}\right]}^{2}}}\right]}^{2},$$

where f(x,y) is the gray value of the center point (x,y) of the subset before deformation and *g*(*x*′, *y*′) is the gray value of the center point $\left(x^{\prime },y^{\prime }\right)$ of the subset after deformation. $f_{m}$ and $g_{m}$ are the average gray values of the subset before and after the deformation:

$$f_{m}=\frac{1}{{\left(2M+1\right)}^{2}}\sum_{x=-M}^{M}\sum_{y=-M}^{M}f(x,y),$$

$$g_{m}=\frac{1}{{\left(2M+1\right)}^{2}}\sum_{x=-M}^{M}\sum_{y=-M}^{M}g\left(x^{\prime },y^{\prime }\right)$$

It was reasonable to assume that the shape of the reference square subset was changed in the deformed image. However, based on the assumption of the deformation continuity of a deformable entity, a set of adjacent points in the reference subset were still regarded as the adjacent points in the target subset. As shown in Figure 3, the coordinates of point $Q(x_{i},y_{j})$ around the subset center $P(x_{0},y_{0})$ in the reference subset could be mapped to point $Q^{,}(x_{i}^{,},y_{j}^{,})$ in the deformed subset according to shape function. During the experiment, the rigid body translation of a subset was always accompanied with the expansion and contraction deformation, so the first-order shape function was adopted. The coordinates of point $Q^{,}(x_{i}^{,},y_{j}^{,})$ were calculated by the following shape function:

$$x_{i}^{,}=x_{i}+\mu +\frac{\partial \mu }{\partial x}\Delta x+\frac{\partial \mu }{\partial y}\Delta y,$$

$$y_{j}^{,}=y_{j}+\upsilon +\frac{\partial \upsilon }{\partial x}\Delta x+\frac{\partial \upsilon }{\partial y}\Delta y,$$

where $\Delta x=x_{0}-x_{0}^{,},\Delta y=y_{0}-y_{0}^{,}$, μ and υ are the x and y direction displacements of the center point of the reference facet, and $\frac{\partial \mu }{\partial x}$, $\frac{\partial \mu }{\partial y}$, $\frac{\partial \upsilon }{\partial x}$ and $\frac{\partial \upsilon }{\partial y}$ are the first-order displacement gradients. 

In this test, as Figure 2 shows, images of the treated front surfaces of specimens during the compression test were taken by a digital camera with a resolution of 5472 × 3648 pixels. In order to avoid undesired shadows in the image, a white light was applied in this test. During the whole testing process, the frequency of the images collected by the camera was consistent with that of the YHD displacement meter recording system. The image of the sample was used as input data for the 2D-DIC software to determine the displacement of each point on it. The total area researched by DIC is equal to 150 × 200 mm^2^. DIC analysis considers the subset and step size set as 51 and 9 pixels, respectively. 

## 3. Experimental Results

### 3.1. Physical Properties of Concrete after Exposure to High Temperatures

**Figure 4:**
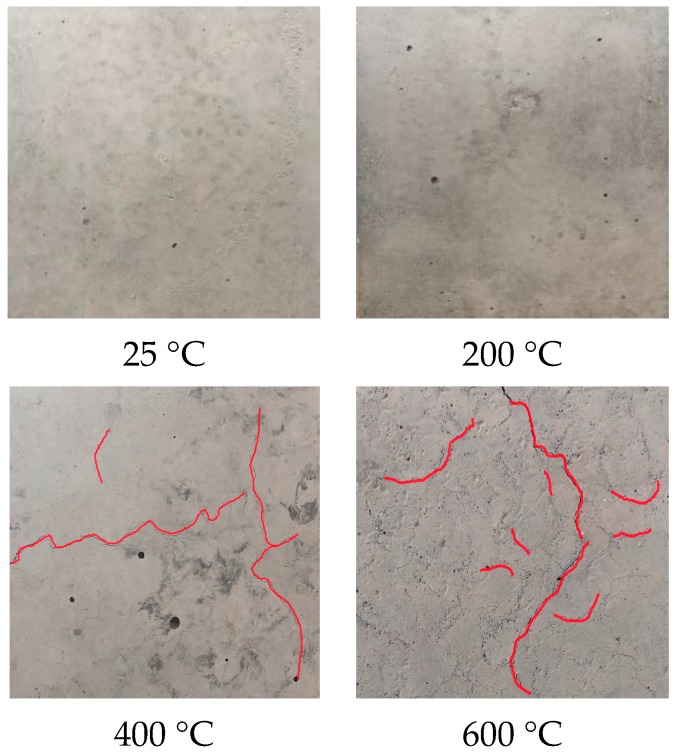
Surface texture of the concrete samples exposed to different temperatures.

By observing the concrete surface, damage to the concrete after exposure to elevated temperatures can be roughly detected. The assessment of fire damage concrete usually begins with visual observation of color changes, cracking and spalling on the concrete surface. Figure 4 illustrates the specimens of the C-1 group after exposure to different temperature levels (200 °C, 400 °C, 600 °C), along with one at ambient temperature. After exposure to 200 °C, there was no visible change in the appearance of the specimens. However, when the specimens were exposed to 400 °C, some light micro cracks began to appear on the surface of the concrete, and the color of the specimens also changed to grey-white. The fine fractures were caused by the decomposition of calcium silicate hydrate (CSH) and the thermal expansion of cross aggregates. When the temperature increased to 600 °C, the color of specimens turned to a slightly pink reddish color, and the cracks became more obvious. During this temperature range, calcium hydroxide (Ca(OH)_2_)—which is the pivotal element in cement paste—decomposed and caused a dry shrinkage of the cement paste. This discoloration can be explained by the presence of iron compounds, which dehydrate or oxidize in this temperature range.

**Figure 5:**
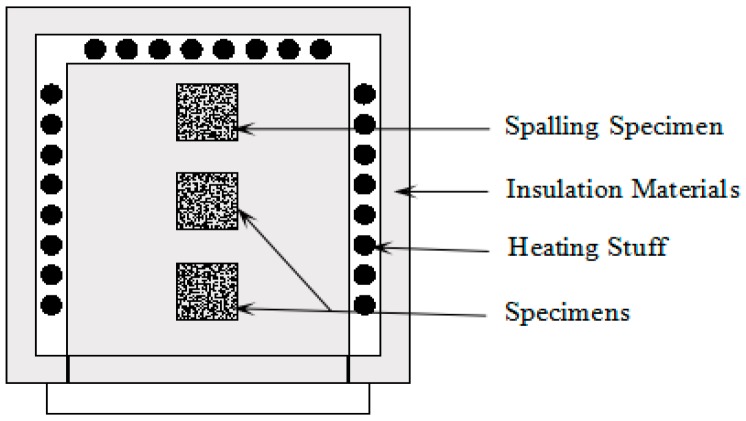
Section drawing of the furnace.

The spalling of concrete generally occurs when it is exposed to elevated temperatures or mixed by lower w/c ratios. When the temperature is below 300 °C or the w/c ratio is higher than 0.32, the spalling of concrete rarely occurs [36]. When subjected to elevated temperatures, many randomly distributed cracks were formed inside and on the surface of the concrete. The occurrence of spalling depends on whether the crack space is sufficient enough to release the pore pressure [37]. When the pore pressure cannot be completely released in the crack, the concrete will spall after reaching the critical strength condition [38,39]. A spalling occurred during this experiment, which can be attributed to two points. First, the w/c ratio of this specimen was just 0.34, resulting in a denser internal structure of concrete. When exposed to an elevated temperature, it is difficult for vapor to move out from concrete into the atmosphere. The vapor pressure, which causes cracks in concrete, comes into being. When the temperature came to a certain level, the tensile stress of the concrete could not resist the pore pressure, and the spalling of concrete occurred. Another cause was that the specimen was located at the innermost part of the furnace, as shown in Figure 5. Thus, its surface temperature was higher than the other two specimens. 

**Figure 6:**
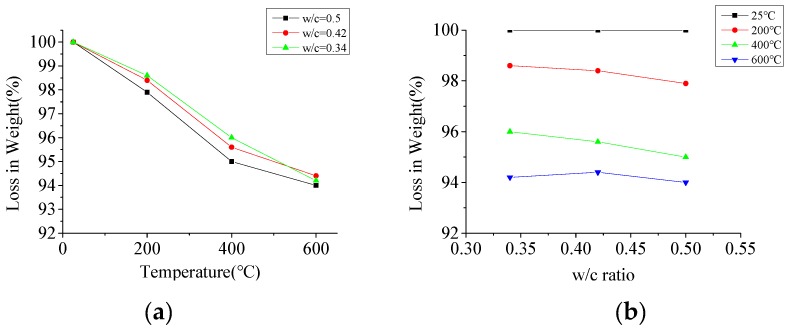
The weight loss of the specimens; (**a**) the effect of elevated temperatures on the weight loss, (**b**) the effect of w/c ratio on the weight loss.

In order to study the effect of high temperature on the weight loss of concrete, the specimens were weighed before being placed in the furnace and after cooling to ambient temperature. The electronic scale with an accuracy of 0.1 g was used to weigh the specimens. Figure 6a shows the change in the weight loss of the specimens after exposure to different temperatures. When exposed to 200 °C and 600 °C, the weight loss ratios were about 2% and 6%, respectively, but it was shown that the largest drop in temperature was between 200 °C and 400 °C. At a temperature of about 200 °C, weight loss was mainly caused by the evaporation of free water in the outer part of the concrete. There was a sudden increase in weight loss at a temperature of about 400 °C. When exposed to a temperature of 400 °C, the inner part of the concrete lost its free water, and the outer part of the concrete lost its chemically-bounded water. Hence, the cumulative effects of these parameters made specimens express considerable amounts of weight loss at elevated temperatures.

Figure 6b shows the effect of the w/c ratio on the weight loss of the concrete specimens. From Figure 6b it can be observed that as the w/c ratio increased, there was an increase in the percentage of weight loss within 400 °C. After heating to 600 °C, there were no obvious differences among the three types concretes. This can be attributed to the fact that weight loss below 400 °C is mainly due to the evaporation of water, and above 400 °C is mainly due to the decomposition of C–S–H and (Ca(OH)_2_) [40]. From the observation above, it can be found that the w/c ratio had less effect on weight loss than temperature.

### 3.2. Mechanical Properties of Concrete after Exposure to High Temperatures

#### 3.2.1. Failure Modes

**Figure 7:**
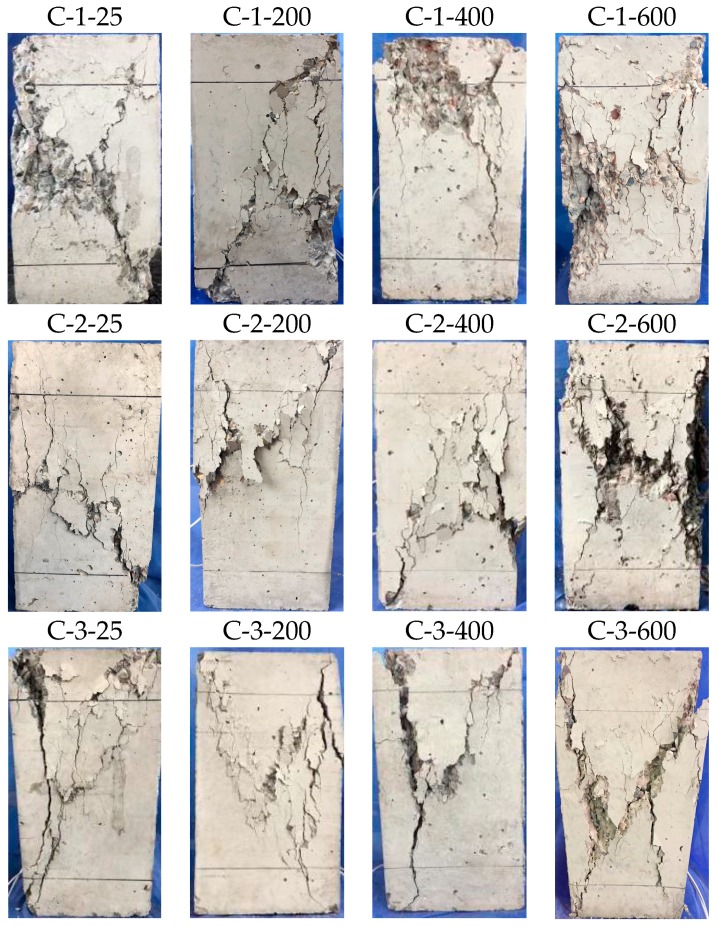
Failure modes of the compression test.

Failure modes of three different w/c ratio specimens after the compressive loading are shown in Figure 7. As can be seen, the damage progression and failure mode of the specimens’ exposure to elevated temperatures was different from reference specimens.

In the case of the C-1 group, when the mechanical load reached about 70% of the peak value, cracks on the reference specimen began to appear near the top left corner. With the load increase, the cracks continued to grow, and some new, fine cracks appeared. They then developed towards the specimens’ end, producing a slant crack map. At the descending stage, several small pieces of concrete peeled off. At temperatures of about 200 °C and 400 °C, the effect of elevated temperature on the sequence of failure progression was not significant, except that the bearing capacity decreased slowly. When exposed to a temperature of 600 °C, more fine cracks appeared on the concrete surface, and concrete spalled off at an early period. The same situation can be observed in the C-2 and C-3 groups, as shown in Figure 7.

In the case of specimen exposure to the same temperature, the damage of the lower w/c ratio specimens were more serious. At a temperature of about 600 °C, the failure modes of the C-3-600 specimen had a significant difference compared with the C-1-600 and C-2-600 specimens, as shown in the rightmost three photographs in Figure 7. These phenomena can also be observed at 200 °C and 400 °C, but were not so obvious as that at 600 °C. After exposure to higher temperatures, micro cracks formed along the aggregate-mortar interfaces. The damage was more obvious at lower w/c ratio specimen, resulting in serious disintegration of the C-3 group which was cast with low w/c ratio.

#### 3.2.2. The Results of the DIC Method

**Figure 8:**
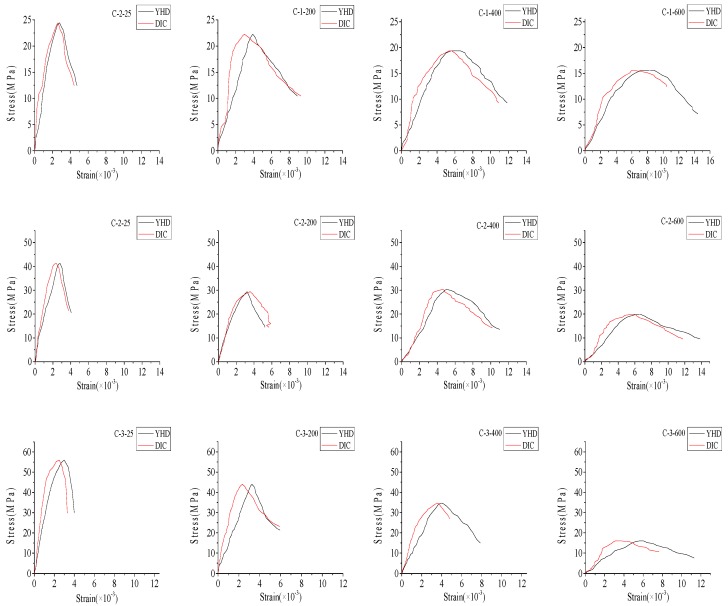
Stress–strain curves of specimens at different temperatures.

The compressive stress–strain curves for different specimens are shown in Figure 8. The values of strain were observed from the DIC and YHD displacement meter, and the values of load were obtained from the compression testing machine. For the three types of concrete, with the temperature increase, the peak stress decreased and the corresponding peak strain increased. Factors that affect the stress–strain curves of concrete at elevated temperatures are the initiation and propagation of micro-cracks and the disintegration of calcium silicate hydrates. These factors caused a rapid decrease in the compression strength and a large increase in strain above 400 °C. 

In Figure 8, the black line represents the result of the YHD displacement meter, and the red line represents the result calculated by DIC. Out of the three samples tested, the test result of the specimen which replicated the average behavior was used for the comparison of behavior with respect to different temperatures. By comparing the results of DIC and YHD displacement meters, we found that they were in good agreement in the elastic phase, and the difference between them increased gradually with the increase of pressure at all specimens. This phenomenon was evident in group C-3, and the largest difference (40%) in peak strain—measured by the displacement meter and DIC—occurred in this group. Because of the dense internal structure, more cracks were formed after exposure to elevated temperature, and the surface concrete was more prone to fall off during the loading process. 

**Figure 9:**
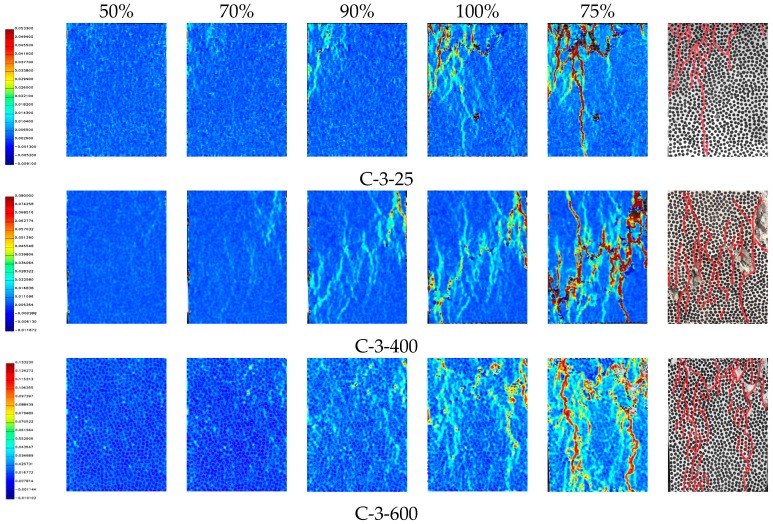
Distribution of principal strain.

Figure 9 below shows the full field strain distribution of the specimen subjected to various temperatures at different load levels. From left to right, the principal strain distribution of 50%, 70%, 90%, 100% and 75% (in the descending stage) of the peak bearing capacity are presented in the figure. DIC technology can obtain the strain of the specimen surface in the full-filled distribution at any stage of the loading process. When the temperature was below 400 °C, the distribution of the surface strain of the concrete had obvious regularity. The DIC technique can clearly draw the high tension areas, even if no cracks were formed on the surface. With the increase of pressure, these regions gradually formed cracks and even concrete spalling. When heated to 600 °C, the strain distribution on the concrete surface was sporadic. It can be seen that temperature has a serious and irreversible effect on concrete. However, the strain distribution at this region cannot be provided when the surface concrete falls off, as shown in Figure 9 for the C-3-600 specimen. This may also limit the range of application of DIC.

#### 3.2.3. Residual Compressive Strength

**Figure 10:**
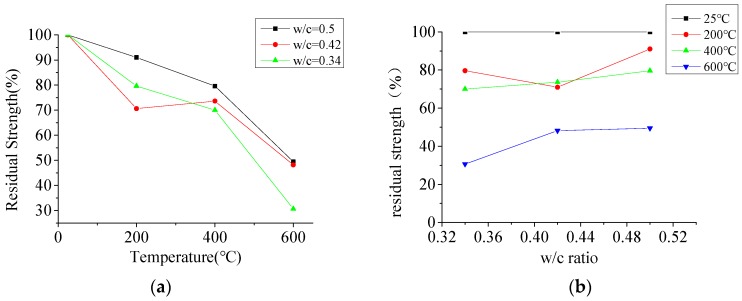
The residual compressive strength of specimens; (**a**) the effect of temperature on the compressive strength, (**b**) the effect of w/c ratio on the compressive strength.

Figure 10a shows the effect of temperature on the compressive strength of specimens. After exposure to elevated temperature, the compressive strength of concrete decreased with the temperature increase, which could be attributed to the dehydration of concrete by the driving out of free water and fraction water of the hydration of concrete. When the temperature came to 600 °C, calcium hydroxide (Ca(OH)_2_)—which is one of the most important compounds in cement paste—decomposed, partially resulting in the shrinkage of cement paste. Cement paste is an important link between coarse aggregate, which is the strength-giving compound of concrete. As a result of the shrinkage of cement paste, many cracks were formed between coarse aggregates inside the concrete, resulting in a 50 to 70% reduction in the compressive strength of concrete. Most of the changes that concrete experiences at this temperature level are considered irreversible. It should be noted that the shrinkage of cement paste and the expansion of aggregate plays an important role in the reduction of the compressive strength of concrete after exposure to elevated temperatures.

In Figure 10a, it can be observed that the compressive strength of concrete decreased with a temperature increase, except for the C-2-400 specimen, whose residual compressive strength increased after exposure to 400 °C. This situation also occurred in other studies. This can be attributed to the loss of free water in the concrete, leading to the shrinking in gel component, which enhances the bond between the cement paste and the aggregate.

Figure 10b shows the effect of the w/c ratio on the compressive strength of specimens. When the temperature was below 400 °C, the influence of the w/c ratio on the residual bearing capacity was not obvious, but it was obvious when the temperature rose to 600 °C. The maximum difference of bearing capacity between specimen C-3-600 and specimen C-1-600 increased up to 18%. The main reason for this phenomenon was that the low w/c ratio caused the dense microstructure of the specimen, which did not allow the release of pressure caused by evaporation of water under the elevated temperature. In this temperature range, the decomposition of calcium hydroxide (Ca(OH)_2_) further increased the pore pressure. These factors led to a greater loss of compressive strength in specimens with smaller w/c ratios.

#### 3.2.4. The Effect of Temperature on the Elastic Modulus

**Figure 11:**
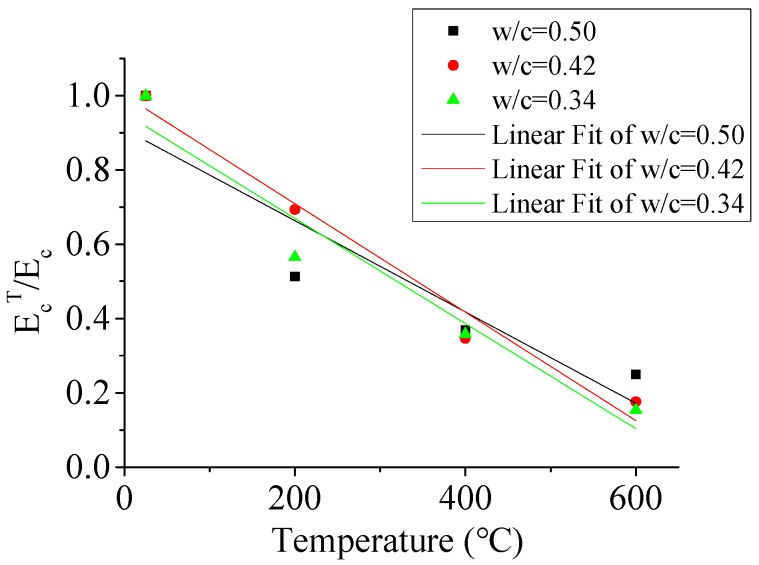
Elastic modulus of specimens after exposure to different temperatures.

Figure 11 shows the effect of the w/c ratio and temperature on the elastic modulus. The values of the elastic modulus of specimens were the secant modulus at 40% of the peak stress from the experimental compressive stress–strain curve of YHD. It can be seen from Figure 11 that the trends in the loss of relative elastic modulus (E_c_^T^/E_c_) with and increasing temperature were similar in the three different w/c ratio specimens. When the temperature reached 600 °C, the relative elastic moduli were about 20% of the original unheated specimens. The effect of the w/c ratio on the elastic modulus of concrete was obvious after heating to 200 °C. The different w/c ratios resulted in different free water content, causing various void pressures at the concrete interiors under elevated temperatures, which caused many fine cracks. When the temperature reached 400 °C or above, the effect of the w/c on the elastic modulus was not obvious. In this temperature range, the decrease in the elastic modulus was mainly due to the deterioration of the microstructure and chemical changes, which can be used to explain the above phenomenon.

## 4. Conclusions

The axial compression tests on ordinary concrete were carried on using a servo-hydraulic testing machine. The crack development process based on DIC technology was monitored. The following conclusions can be drawn from the experimental results of this research:

1. Concrete with different w/c ratios present similar colors and cracking when exposed to the same temperature. The deterioration of concrete performance occurs after exposure to 400 °C or above. After heating to the same temperature, the specimens with low w/c ratio were easy to crack, especially beyond 400 °C. As for the specimens with the same w/c ratio, with a temperature increase, concrete cracked at the early loading stage.
2. The residual compressive strength at 200 °C still retained about 85% of the original unheated value, however, the values at 400 and 600 °C reduced to 75% and 45% on average, respectively. The degeneration of compressive strength mainly occurred between 400 °C and 600 °C. The decrease of compressive strength in this temperature range accounted for more than 50% of the total decrease. The effect of the w/c ratio on compressive strength was most obvious at 600 °C. The effect of elevated temperature on the elastic modulus of concrete was more obvious than that on the compressive strength. After heating to 600 °C, the decreased value of elastic modulus was about 80% of the control specimen.
3. In this research, the average error between the result of DIC technology and YHD displacement meter was about 14%. However, from Figure 8 we can observe that the DIC technology and YHD displacement meter had a good agreement, which suggests that DIC technology was an accurate and efficient measurement tool for monitoring displacement and strain fields during the whole loading process. DIC technology can obtain multi-direction field strains on concrete surfaces, which is extremely difficult to obtain by using traditional methods. The results of DIC give an insight on how the stresses acted on concrete during the entire loading process.

## Figures and Tables

**Table 1:** The details of specimens.

Group	Specimen	Coarse Aggregate	Sand	Cement	Water–Cement Ratio (w/c)	Exposure Temperature (°C)
C-1	C-1-25	1264	542	380	0.50	25
	C-1-200	1264	542	380	0.50	200
	C-1-400	1264	542	380	0.50	400
	C-1-600	1264	542	380	0.50	600
C-2	C-2-25	1242	558	420	0.42	25
	C-2-200	1242	558	420	0.42	200
	C-2-400	1242	558	420	0.42	400
	C-2-600	1242	558	420	0.42	600
C-3	C-3-25	1252	512	460	0.34	25
	C-3-200	1252	512	460	0.34	200
	C-3-400	1252	512	460	0.34	400
	C-3-600	1252	512	460	0.34	600
